# Primary leiomyosarcoma of the right atrium: a case report and literature update

**DOI:** 10.1186/1749-8090-5-80

**Published:** 2010-10-12

**Authors:** Haralabos Parissis, Mohamad Taukeer Akbar, Vincent Young

**Affiliations:** 1 Cardiothoracic Department, Royal Victoria Hospital, Grosvernor Rd, Belfast, BT12 6BA, Northern Ireland; 2 Cardiothoracic Department, Basildon & Thurrock University Hospital NHS FT, Essex, UK; 3 Cardiothoracic Department, St James Hospital, Dublin 8, Dublin, Ireland

## Abstract

Leiomyosarcoma of the right atrium is a very rare cardiac tumor. Various combinations of treatments including resection or transplant surgery and Chemotherapy have been advocated. We report a case of a man who presented with pulmonary embolism secondary to right atrial leiomyosarcoma. He was managed by excision of the tumor and reconstruction of the right atrium with autologous pericardium. Postoperatively tumor dissemination was controlled with adjuvant chemotherapy.

A vigorous attempt aiming at tumor clearance followed by adjuvant multimodality therapy along with a tumor surveillance program may improve survival.

## Introduction

Primary cardiac malignancies (PCM) are rare. The prevalence of primary cardiac malignancies has been estimated at only 0.001% - 0.28% [1]. Primary cardiac tumors are detected in 1 in a 1000 autopsies and PCM are found in only about 0.0017% of autopsies [2,3]. Metastatic cardiac tumors are a 100-fold more common than primary lesions. The majority of Primary Cardiac tumors are benign (with half of them being myxomas) [4] and approximately 25% of primary cardiac neoplasms are malignant. Among malignant primary cardiac tumors, the most reported are those histopathologically considered as undifferentiated, followed by angiosarcomas with leiomyosarcomas being rare. Due to delayed presentation there is infrequently, a systemic spread at the time of diagnosis. As a result management of this condition is difficult and controversial.

## Case Report

**Figure 1:**
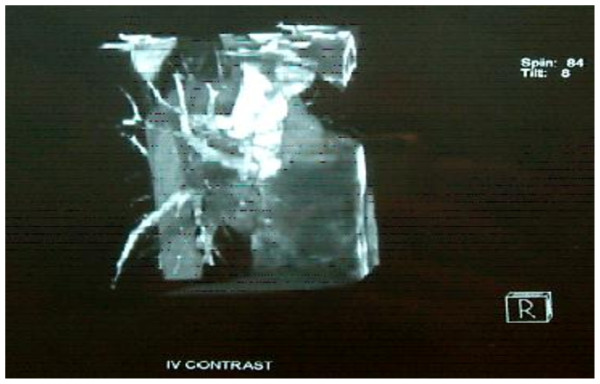
**Three dimensional reconstruction of Computerized Tomogram Pulmonary Angiogram demonstrating large filling defect in the branches of right pulmonary artery**.

**Figure 2:**
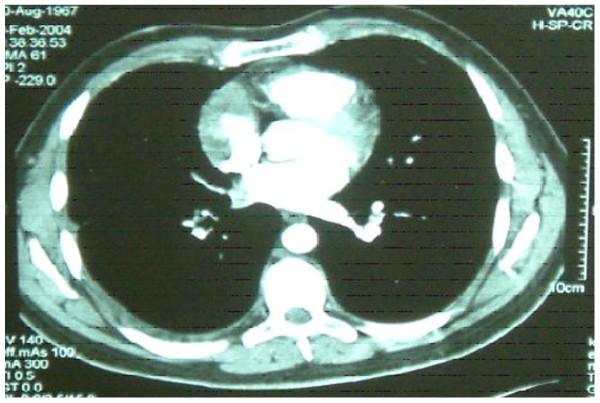
**Intravenous contrast enhanced Computerized Tomogram demonstrating right atrial wall tumor which appears to be lobulated, irregular and of low attenuation**.

**Figure 3:**
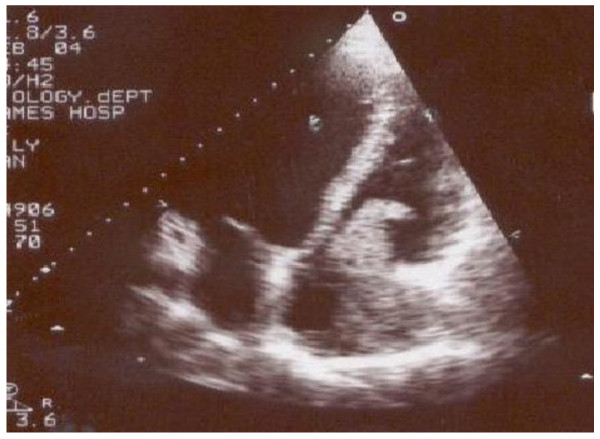
**Transthoracic Echocardiogram demonstrating right atrial tumor extending from the free wall to the tricuspid valve and protruding through it to the right ventricle**.

**Figure 4:**
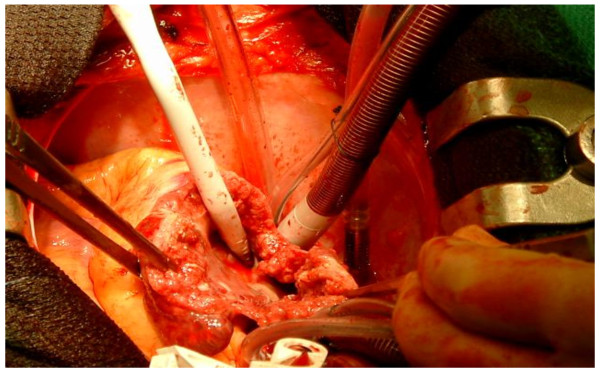
**Right atrial tumor resection**.

**Figure 5:**
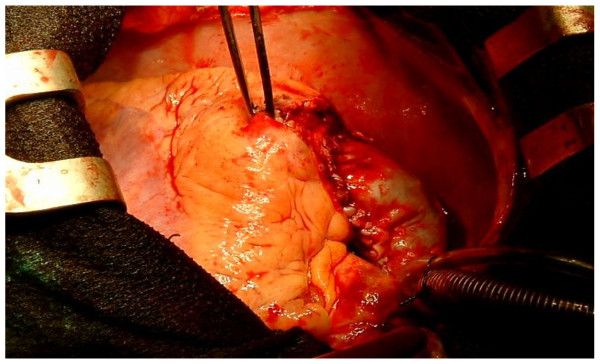
**Right atrial reconstruction with autologous pericardium**.

We present a case of a 36 year old male who was admitted with recent onset of shortness of breath. CT pulmonary angiogram demonstrated large right sided pulmonary emboli (Figure 1). Moreover, a filling defect was noticed in the right atrium (Figure 2). The defect appeared to be lobulated, irregular, of low attenuation and arising from the free atrial wall. On transthoracic echocardiography (Figure 3) the mass was demonstrated to be extending through the tricuspid valve. A presumptive diagnosis of right atrial myxoma with complicating pulmonary embolism was made. Urgent surgery was arranged. At the time of surgery the right atrial appendage was noted to be very congested and "angry looking". Total cardiopulmonary bypass was established using aortic and bi-caval cannulation. The right atrial cavity was found to be replaced by a friable tumor which had "fronds like" appearance (Figure 4). The mass was extending through the tricuspid valve to the right ventricle. A sample of the tumor was subjected to frozen section examination which suggested the diagnosis of Leiomyosarcoma. The entire free wall of right atrium was excised extending from and to the origin of vena cavae. Anteriorly the incision was carried forward up to the atrioventricular groove, taking care to preserve the right coronary artery while ensuring macroscopic clearance of tumor. The resection margins were submitted for histological examination and were subsequently proven to be tumor free. The right atrium was reconstructed using autologous pericardium (Figure 5). Bilateral pulmonary embolectomy was also performed. Histological examination of tumor confirmed the frozen section findings. On the cut surface, the tumor had a whirled white appearance, with focal brown areas. The microscopic examination revealed the presence of a spindle cell tumor, forming fascicles orientated at right angles. The study revealed the morphological aspect characteristic to leiomyosarcoma.

Although resection margins were clear the margin width was deemed to be inadequate. After recovery from surgery the patient was submitted to chemotherapy with Doxirubicin and Isofomaide. A tumor surveillance plan involving serial echocardiograms is planned.

## Discussion

Of the few hundred cases of malignant tumors of the heart reported, most have been based on autopsy series. Moreover, throughout the last 30 years literature from a 100 plus articles on cardiac neoplasms, only few publications are reporting on primary cardiac malignancies [5].

Cardiac sarcomas are the second most common type of primary cardiac neoplasms with Leiomyosarcomas to consist of 8% of cardiac sarcomas [6,7]. As per Kim et al [8] angiosarcomas and unclassified sarcomas are the most common sarcomas of the heart accounting for 76%of the cases, with leiomyosarcomas being a minority. There is a wide age and size range with a slight female predilection. As per Zhang et al [6] the sarcoma arises in the atria/pulmonary vessels in 74% of the cases, and in the ventricles, mitral valve, and epi/pericardium in 14%, 3.7% and 7.4% of the cases correspondingly. There is probably a slight left side predilection. In contrary, predominantly right side distribution is given in other reports [8] with right atrial involvement being 58% and left atrial 25% of the cases.

Leiomyosarcoma favors the left atrium and most likely originates from pulmonary vein smooth muscles and present as a left atrial tumor. Affected patients typically present in the 4th decade of life which is slightly younger than the average age at presentation for cardiac sarcoma patients. Unlike angiosarcomas hemorrhage is unusual. Those tumors are likely to involve the mitral valve and extend into the pulmonary veins and therefore present with pulmonary congestion. Macroscopically the tumor appears as gelatinous mass and maybe multiple in up to 30% of the cases [9].

In our case report the site of origin of the tumor was the right atrium which is rare. The presentation was consistent with thromboembolic phenomenon commonly associated with this tumor site. Other possible presentations include progressive or sudden right sided cardiac failure on the account of tricuspid valve blockage by the tumor or paroxysmal atrial arrhythmias. Broadly speaking, cardiac tumors produce a large variety of symptoms through any of 4 mechanisms. Their mass can obstruct intracardiac blood flow or interfere with valve function. Local invasion can lead to arrhythmias or pericardial effusions with tamponade. Bits of tumor can embolize, causing systemic deficits when the tumors are on the left side of the heart. Finally, the tumors may cause systemic or constitutional symptoms.

Echocardiographic imaging is the most sensitive imaging technique with ability to identify tumors as small as 3 mm. However, soft-tissue characterization remains limited compared with that achieved with computed tomography (CT) and magnetic resonance (MR) imaging, and myocardial disease such as tumor infiltration is not clearly depicted [9]. On the other hand with MRI or contrast enhanced CT the tumor has to be around 1 cm in size before becoming detectable.

In this case CT scan raised the suspicion of intracardiac tumor by depicting a low attenuation filling defect and echocardiography confirmed the diagnosis. A preoperative tissue diagnosis was not attempted due to the emergent presentation. However atypical appearance of the right atrium and of the tumor raised the suspicion of malignancy and frozen section examination was confirmatory. It has been recommended that all atrial tumors should be subjected to frozen section examination in order to ensure optimum surgical resection.

According to Mayer et al[10] half the patients with cardiac sarcomas, are presented with high grade tumors and distant metastases: lungs 35.7%, lymph nodes 14.2%, and liver 7.14%. Tumor spread from primary cardiac sarcoma to the bone is very rare and has a poor prognosis. Only six cases have been reported in the literature [11]. Furthermore, from the patients that are deemed suitable for surgery, complete macroscopic resection is only possible in 33% [12].

Operative mortality has been reported to be high at 8.3% with an overall actuarial survival of 14% at 24 months after resection [13]. Likewise other groups [8,12] have reported poor prognosis with a median survival time of 25 months after diagnosis.

As per Burke et al [14], the survival rate on univariate analysis was more favorable for patients with tumors located on the left side of the heart, without necrosis, with a low mitotic count, and without metastasis at diagnosis. By multivariate analysis, a low level of mitotic activity and any therapy were the only significant factors affecting survival rate. Furthermore tumor grade, unlike histological type, appears to be prognostically important in cardiac sarcoma [6].

The optimum treatment of Leiomyosarcoma is not known. Of the several reports in the literature, patients subjected to multimodality treatment including heart transplantation (The most common cause of death is local recurrence of the tumors in 50% of the cases [12]) have longer survival.

We adopted a strategy that would ensure local control of the tumor by surgical resection and address systemic spread by adjuvant chemotherapy. Tumor shrinkage can be achieved by chemotherapy prior to surgery in non emergency setting. Given the high risk of tumor recurrence we plan to follow-up the patient with serial echocardiographic scans with the view to further surgical or chemotherapeutic intervention aimed at early treatment of recurrence.

## Conclusion

In conclusion PCM are rare and will always pose a diagnostic dilemma. Nevertheless, atypical presentation of suspected "atrial myxoma" should raise the possibility of rare atrial tumors. Unfortunately, almost half of those tumors have metastasized at presentation, up to 30% could be multifocal and the rest may be amenable to surgery. All such tumors should be subjected to frozen section examination intraoperatively.

Surgery carries a high mortality and the overall long survival was only achieved in patients who survived the initial surgery well.

## Consent

Written informed consent was obtained from the patient for publication of this case report and accompanying images. A copy of the written consent is available for the review by the Editor-in-Chief of this journal.

## Competing interests

The authors declare that they have no competing interests.

## Authors' contributions

HP conceived of the study and wrote the manuscript with the help of MTA. VY overlooked the progress of the manuscript and advised on valuable points. All authors read and approved the final manuscript.
